# Inflammatory Bowel Disease: An Indication to Screen for Thrombophilia?

**DOI:** 10.3390/diseases10010014

**Published:** 2022-02-23

**Authors:** Nour M. Moukalled, Jana G. Hashash, Ali T. Taher

**Affiliations:** 1 Division of Hematology and Oncology, Department of Internal Medicine, Naef K. Basile Cancer Institute, American University of Beirut Medical Center, P.O. Box 11-0236, Riad El Solh, Beirut 1107 2020, Lebanon; ataher@aub.edu.lb; 2 Department of Gastroenterology and Hepatology, Mayo Clinic, Jacksonville, FL 32224, USA; jhashash@gmail.com

**Keywords:** inflammatory bowel disease, thrombosis, hereditary thrombophilia

## Abstract

Inflammatory bowel diseases (IBD) are systemic conditions characterized by multiple intestinal and extra-intestinal manifestations related to the associated chronic inflammatory state. Among their diverse extra-intestinal complications, venous thromboembolism (VTE) remains one of the most under recognized causes of morbidity and mortality in these patients, highlighting the need for a better understanding of the underlying mechanism of hypercoagulability, in addition to the role of acquired and inherited risk factors that further increase the risk of thrombosis with its impact on patients’ outcomes. We hereby present a review of the data regarding thrombosis in the setting of IBD, elucidating the possible role for screening in this high-risk category of patients and specifically in areas where inherited thrombophilia is expected to be highly prevalent, reporting two patients with IBD, one who developed a cerebrovascular event and another one who had recurrent VTE events; nevertheless, both of them had inherited thrombophilic mutations. The identification of specific genetic abnormalities in those patients reintroduces the controversy related to the need to screen a specific category of patients with IBD for hereditary thrombophilia, especially in regions characterized by a higher prevalence of such thrombophilic alterations.

## 1. Introduction

Inflammatory bowel diseases (IBD) are inflammatory conditions of the gastrointestinal tract which are categorized into ulcerative colitis (UC) and Crohn’s disease (CD). These two subtypes are differentiated by unique features related to the anatomic site and pattern of disease involvement; as well as the depth of the ulcers noted on endoscopy. Nonetheless, both conditions share similar systemic manifestations related to chronic intestinal and extra-intestinal inflammation [1]. Among the multiple extra-intestinal complications of IBD, venous thromboembolism (VTE), which mainly includes lower extremity deep vein thrombosis (DVT) and pulmonary embolism (PE) [2], remains one of the most underrecognized causes of morbidity and mortality in this patient population [3]. Other less commonly reported sites of thrombosis include cerebral, cardiac, hepatic, mesenteric, and retinal veins and arteries [4]. IBD patients are at risk for venous and less commonly arterial thrombosis, even when the IBD is quiescent, with almost a three-fold higher incidence rate compared to the general population [5,6,7]. This rate of VTE increases by approximately 8-fold in patients with active IBD [8]. Grip et al. reported that IBD patients developed VTE earlier in life when compared to a controlled non-IBD population [9]. The rate of VTE events has been reported to be 1.5–7.7% in previous trials [4], with a mortality rate of 25% per event [5], despite the fact that this incidence approaches 40% in post-mortem evaluations [4]. The underlying mechanism leading to the hypercoagulable state in IBD patients remains to be unraveled. Multiple hypotheses have attributed this increased risk of thrombosis to qualitative and quantitative defects in the hemostatic system, including the pro-coagulation (elevated factor V and lipoprotein-(a) among others), anti-coagulation (reduced levels of protein C and S), as well as the fibrinolytic pathway (decreased tissue plasminogen activator) [1], platelet, and endothelial abnormalities [10]. Papa et al. have shown that IBD patients have a greater thickness of the carotid intima-media, which correlates with early atherosclerosis [11]. Acquired risk factors are thought to play the most important role in thromboembolic events complicating IBD. Nonetheless, recent reports have focused on the prevalence of inherited thrombophilic conditions and their role in the pathogenesis of thromboembolism in IBD patients. This has led to the controversial discussion related to the need to screen such patients for genetic mutations that would further increase their risk for developing VTE events, especially in areas characterized by high prevalence of such inherited abnormalities, including the Mediterranean region. We hereby include a brief literature review of the available data regarding thrombosis in the setting of IBD, based on a search using PubMed conducted 1998 to 2020 using the following key words: inflammatory bowel disease, thromboembolism, and inherited thrombophilia (additional references identified from a review of citations), elucidating the possible role for screening in this high risk category of patients and specifically in areas where inherited thrombophilia is expected to be highly prevalent, utilizing our experience with two patients with identified thrombophilic mutations in the setting of IBD, one with a cerebrovascular event after recurrent disease flares and another with a VTE event during a disease flare up.

## 2. Cases

**Figure 1:**
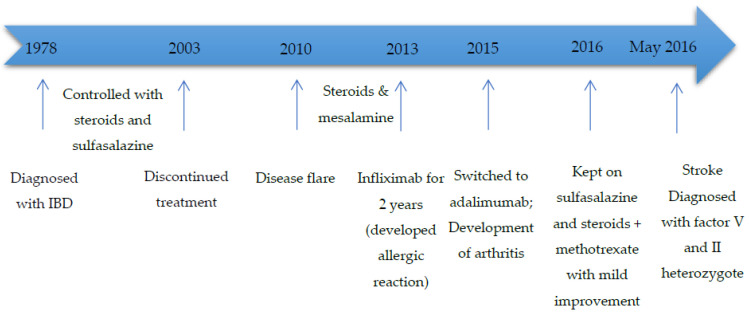
Summary of events for case I. IBD: inflammatory bowel diseases.

Case I is a 49-year-old Lebanese female patient with a history of UC since the age of 11 years and hypertension who presented for evaluation of dizziness, gait imbalance, diplopia, and dysarthria. She had previously received different treatments for her colitis (as indicated in Figure 1). Her disease course was complicated by tenosynovitis, arthritis of the ankles and wrists, in May 2015. A year later, in May 2016, she complained of dizziness, gait imbalance, diplopia, and dysarthria. She also had erythematous skin lesions consistent with erythema nodosum. Laboratory investigations revealed mild leukocytosis (white blood cell count (WBC) 11.7 × 10^9^/L, absolute neutrophil count (ANC) 9.945 × 10^9^/L), thrombocytosis (platelets 527 × 10^9^/L), anemia (hemoglobin 112 g/L), and a normal metabolic profile. She had vitamin B12 deficiency (141 pg/mL) with a normal folate level. Prothrombin and activated partial thromboplastin time were normal. C-reactive protein was normal (0.9 mg/L), ESR (erythrocyte sedimentation rate) 26 mm/h, while Brucella direct and indirect antibodies were negative. Borrelia antibodies were negative. Cerebrospinal fluid studies showed normal glucose level (48 mg/dL), negative bacterial cultures, and normal oligoclonal band IgG index. Herpes Simplex virus type 1/2 and Varicella virus were not detected. Anti-HU, NMDA, NMO-Aquaporin, and YO antibodies were all negative. Brain MRI showed restricted diffusion within the medulla diagnosing her with an acute medullary infarct. Cardiac evaluation revealed no arrhythmia or valvular disease. After diagnosing the acute stroke, thrombophilia workup was ordered. Anti-cardiolipin antibodies and lupus anticoagulant were negative. She had normal antithrombin (107%; normal 80–120%) and protein C (105%; normal 70–150%), with a normal protein S (59.1%; normal 60–150%) during the acute event (chromogenic assays). She was found to have heterozygote factor V Leiden (G1691A) and factor II (G20210A) mutations (compound heterozygote). To note that, the thrombophilia panel done at that time included multiple gene mutations, and the patient was additionally found to have homozygote methylenetetrahydrofolate reductase (MTHFR) gene mutation (A1298C), with normal homocysteine level (10.6 µmol/L). She did not have any previous personal or family history of thrombosis or miscarriages (G3P3A0) and had never received hormonal therapy. She was started on clopidogrel 75 mg daily and continued working with physical and speech therapy with gradual improvement of her neurological symptoms. Figure 1 includes a summary of events for this patient since the diagnosis of IBD.

**Figure 2:**
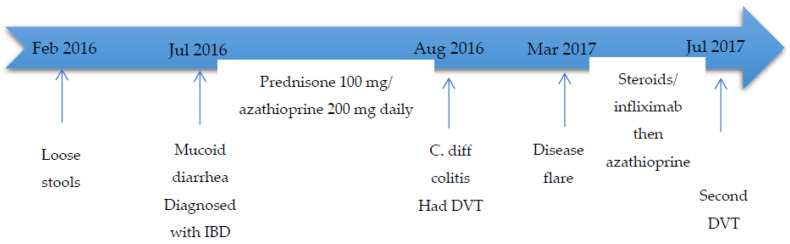
Summary of events for case II. Legends: IBD: inflammatory bowel diseases; C. diff: clostridium difficile; DVT: deep vein thrombosis.

Case II is a 30-year-old Lebanese male patient who developed abdominal pain, loose stools, and intermittent hematochezia, in February 2016, in addition to weight loss of 25 kg over a 6-month period. In July 2016, he had worsening of his symptoms with mucoid bloody diarrhea (more than 20 episodes per day), with no improvement on antibiotics. A colonoscopy at that time revealed diffusely inflamed mucosa less prominent at the level of the recto-sigmoid with some areas of normal looking mucosa (conserved vascular pattern). The mucosa between the sigmoid colon and the cecum appeared congested and edematous with loss of vascular pattern. Additionally, these areas contained scattered superficial ulcerations. The terminal ileum appeared normal. Biopsy showed chronic active colitis characterized by a dense lymphoplasmacytic infiltrate with patchy architectural distortion, favoring IBD colitis. After 4 weeks, he had worsening of his diarrhea, was hospitalized, and was found to have clostridium difficile (C. diff) colitis (positive by polymerase chain reaction). He received a course of oral metronidazole and had a mild improvement in his diarrhea, so he was then given a course of oral vancomycin with significant improvement of his symptoms. During the same period, he developed right lower extremity pain and swelling and was found to have a right lower extremity DVT, which was treated with low molecular weight heparin (1 mg/kg every 12 h) then dabigatran (150 mg twice daily) for 6 months. Once the patient left the hospital, he continued to taper down the steroids and also self-discontinued the azathioprine, without recurrence of any symptoms for 6 months. He remained in clinical remission until March 2017, when he experienced recurrent abdominal pain and bloody diarrhea and was hospitalized for management of an IBD flare. Colonoscopy was performed and showed severe pan-colitis. In July 2017, the patient complained of fatigue and abdominal pain. Infliximab trough level was low (<0.3 μg/mL) with high antibody level (91 ng/mL), so the infliximab was discontinued. He was also found to have another VTE event, involving the left lower extremity, and was then restarted on low molecular weight heparin (1 mg/kg every 12 h). Given the recurrent thrombotic episode, the patient was referred for evaluation of thrombophilia. Laboratory evaluation revealed anemia (hemoglobin 117 g/L), a normal WBC (5.7 × 10^9^/L), a normal platelet count (327 × 10^9^/L), normal antithrombin, and protein C and S. He had heterozygote factor V Leiden and homozygote MTHFR (C677T) mutations (which was also included in the multiple gene mutation panel done at that time). He had normal vitamin B12 and homocysteine levels (9.5 µmol/L), with mild folic acid deficiency (3.7 ng/mL). He did not have any prior personal or family history of thrombosis; however, his mother had two miscarriages during the first trimester, with three successful deliveries without the need for any anticoagulation. He was kept on anticoagulation and started on folic acid 5 mg orally daily. Currently, the patient’s symptoms are well controlled on mesalazine 200 mg daily, and he was shifted to dabigatran (150 mg twice daily). Figure 2 includes a summary of events for the patient.

## 3. Literature Review

Testing for hereditary thrombophilia remains controversial with limited utility in most clinical settings. Recent guidelines by the National Institute for Health and Care Excellence [NICE] recommend considering testing in case of an unprovoked VTE in a patient with a first-degree relative with a documented DVT/PE, when there is a plan to discontinue anticoagulation therapy [NICE], 2020 [12], while the American Society of Hematology (ASH) recommends referral for an expert opinion regarding testing in patients who develop VTE in the setting of a major transient risk factor if they have a positive family history or concurrent exposure to hormonal therapy [13]. There have been some variations across different guidelines; nonetheless, there is a general consensus that most patients with VTE do not benefit from testing for hereditary thrombophilia, which can generally be considered in those with weak provoking factors, a strong family history, or recurrent events at a young age [14].

**Figure 3:**
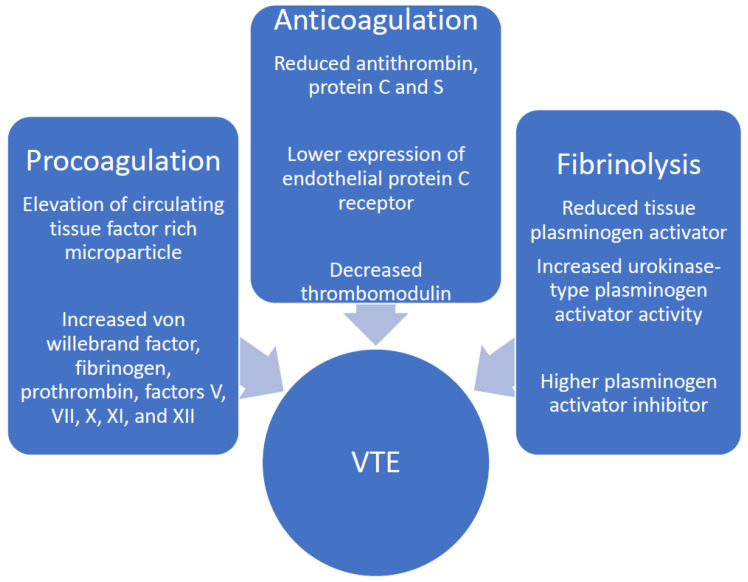
Coagulation abnormalities in IBD. VTE: venous thromboembolism; IBD: inflammatory bowel diseases.

The identification of heterozygote factor V/II mutations in the first case might not explain the early cerebrovascular event; however, it might have altered the management of this patient in terms of applying thromboprophylaxis in the outpatient setting. For the second patient, the identification of heterozygote factor V Leiden would direct thromboprophylaxis decisions with disease flares. The development of DVT/PE leads to significant morbidity and mortality and has been associated with longer hospital stays, as well as higher hospitalization costs, reaching around USD 45,000 in patients from the Nationwide Inpatient Sample between 1998 and 2004 [15]. This necessitates better understanding and control of additional factors that would further increase the thrombosis risk in these patients. The pathogenesis of thrombosis in IBD is complex and is likely due to the interaction of genetic and acquired factors. The significant inflammatory state seen amongst IBD patients activates many coagulation molecules and inhibits fibrinolysis (Figure 3) [16]. In addition, chronic inflammation suppresses the activity of natural anti-coagulants [4] which normally reduce the levels of inflammatory cytokines such as interleukin 6 and tumor necrosis factor [17], leading to reciprocal reinforcement of inflammation by thrombosis. This explains the higher incidence of thrombosis in patients with acute disease exacerbations and more extensive disease involvement, as well as resulting complications including intestinal strictures, fistulae, abscesses, and perforation among multiple other intestinal and extraintestinal manifestations [8,15,18]. This increased risk justifies the recommendations to provide routine thromboprophylaxis for IBD patients who are hospitalized with acute disease flares [19]. Scoville et al. evaluated 204 IBD patients and reported that one third of VTE events were diagnosed in those with a recent flare of whom most required systemic therapy [20]. IBD patients experiencing acute flares were found to have a statistically significant increase in the risk for thrombosis (hazard ratio 8.4) in a prospective cohort study including 13,756 subjects [8]. On the other hand, UC has been associated with a higher risk of VTE as compared to CD, even during phases of disease remission [21,22]. Evaluation of patients with other inflammatory conditions such as rheumatoid arthritis and celiac disease did not reveal an increased risk for thrombosis [4], further elucidating a multifactorial etiology of thromboembolism in IBD patients. Platelet and endothelial abnormalities also play a role in the pathogenesis of both venous and arterial thrombosis. Previous studies have reported elevated platelet counts in IBD patients, in addition to enhanced platelet aggregation [23]. Multiple other risk factors for primary and recurrent VTE events have been identified in IBD patients, including older age, male gender, younger age at the first VTE event, and steroid use [24,25], in addition to traditional VTE risk factors such as hospitalization, recent surgery, malignancy, presence of indwelling catheter, and hormonal therapy among other factors that are frequently present in IBD patients. The role of genetic factors, such as factor V Leiden and G20210A prothrombin mutations in the multifactorial pathogenesis of thrombosis in the setting of IBD has been frequently reviewed and remains controversial. Around 33% of IBD patients with VTE have an identifiable hereditary thrombophilia [26], which is similar to that in the general population. Factor V Leiden, the most common prothrombotic mutation associated with a five-fold increased risk for thrombosis in the heterozygote state, has been identified in around 5% of Caucasian patients and up to 20–30% of patients with VTE [27]. This mutation causes resistance of the activated factor V to degradation by the activated protein C [1]. Thrombotic IBD patients were noted to have higher prevalence of factor V Leiden mutation compared to those with no thrombosis [28]. IBD patients with factor V Leiden mutation have been reported to be at a significantly greater risk of developing thrombosis compared to those without the mutation, as indicated by data from two meta-analyses [29,30]. The prothrombin G20210A mutation is identified in around 2% of the general population and up to 6% in those with VTE [31] but has been closely associated with IBD by some investigators [32]. On the other hand, homozygote MTHFR mutation with the subsequent hyperhomocysteinemia is found in around 10% of the general population [1] but has a controversial role in the development of VTE [33], and thus, related to the growing evidence of its minimal clinical utility, testing for MTHFR polymorphism is currently not recommended routinely [34]. Nonetheless, the prevalence of these genetic abnormalities in IBD patients has varied greatly across studies, and this has been related to the small number of patients included as well as the variable geographic distribution of such mutations [35,36,37]. This might necessitate a different approach to the screening for thrombophilia in IBD patients across different regions of the world. The protein S level for the first patient was taken during the acute event and was almost normal, and thus likely clinically insignificant, and as previous studies have indicated, greatly reduced levels below the lower limit of reference ranges are required to be associated with increased risk of VTE in patients with inherited deficiencies of protein S [38,39].

Previous reports have shown an increased prevalence of multiple thrombophilia related gene mutations in the Lebanese population, with up to 14% having heterozygote factor V Leiden, 11% having homozygote C677T MTHFR mutation, and around 40% having heterozygote C677T MTHFR mutation [40,41]. Moreover, the clustering of more than one genetic abnormality is expected in a population characterized by high rates of consanguinity. This indicates a possible need to evaluate patients with IBD in specific high-risk areas for these mutations in order to identify those with additional predisposing factors that would further increase their risk for VTE, thus altering their prophylaxis and management.

## 4. Conclusions and Future Perspectives

The presence of one or a combination of inherited thrombophilia related genetic mutations might significantly increase the risk of thrombosis in IBD patients who already have an underlying predisposition to VTE, especially during disease flares. Large prospective studies are needed to identify the significance of screening for such abnormalities and the impact of thromboprophylaxis on the outcome of high risk IBD patients specifically.

## Data Availability

Not applicable.
